# Peer-to-Peer Ultra-Wideband Localization for Hands-Free Control of a Human-Guided Smart Stroller

**DOI:** 10.3390/s24154828

**Published:** 2024-07-25

**Authors:** Xiaoxi Zhang, Yang Chen, Modar Hassan, Kenji Suzuki

**Affiliations:** 1 Degree Programs in Systems and Information Engineering, University of Tsukuba, Tsukuba 305-8577, Japan; 2 Institute of Systems and Information Engineering, University of Tsukuba, Tsukuba 305-8577, Japan; chenyang@ai.iit.tsukuba.ac.jp (Y.C.); modar@iit.tsukuba.ac.jp (M.H.); kenji@ieee.org (K.S.)

**Keywords:** peer-to-peer localization, hands-free control, motorized stroller, human–machine interface, human-centered design

## Abstract

We propose a hands-free control system for a human-guided smart stroller. The proposed method uses real-time peer-to-peer localization technology of the human and stroller to realize an intuitive hands-free control system based on the relative position between the human and the stroller. The control method is also based on functional and mechanical safety to ensure the safety of the stroller’s occupant (child) and the pilot (parent) during locomotion. In this paper, first, we present a preliminary investigation of the humans’ preference for the relative position in the context of hands-free guided strollers. Then, we present the control method and a prototype implemented with an electric wheelchair and UWB sensors for localization. We present an experimental evaluation of the proposed method with 14 persons walking with the developed prototype to investigate the usability and soundness of the proposed method compared to a remote joystick and manual operation. The evaluation experiments were conducted in an indoor environment and revealed that the proposed method matches the performance of joystick control but does not perform as well as manual operation. Notably, for female participants, the proposed method significantly surpasses joystick performance and achieves parity with manual operation, which shows its efficacy and potential for a smart stroller. Also, the results revealed that the proposed method significantly decreased the user’s physical load compared to the manual operation. We present discussions on the controllability, usability, task load, and safety features of the proposed method, and conclude this work with a summary assessment.

## 1. Introduction

Baby strollers are essential tools in childcare. Using a stroller offers several advantages to parents, including reducing the load of physically holding the baby, offering a cozy and safe resting spot for the child, and being easy to move. However, manual baby strollers require the users to push or pull the stroller for navigation, which is tiring under long-time usage or in the case of climbing a slope and especially problematic for mothers of multiple children, pregnant mothers, and even senior guardians. Also, manual operation restricts the parent’s ability to perform other tasks while using the stroller. Lightweight and power-assisted baby strollers are designed to reduce parents’ physical load while pushing the standard stroller. However, the situation where parents’ hands are occupied after shopping or a mother of multiple children needs to hold another child’s hand while pushing a stroller, has not yet been well addressed. A smart stroller with a human-following or a human-guided function can reduce the parents’ childcare burden and give them more freedom to move compared to manual operation strollers. Hereafter, we refer to a motorized stroller with a sophisticated control system as a smart stroller and to a motorized stroller with the ability to follow or precede the user as a human-following or -guided smart stroller. Human-following or -guided smart strollers have been investigated in the literature [1,2]. They investigated a smart human-following stroller by utilizing computer vision technology to track the target human and recognize human pose to enable the stroller to follow. However, considerations of relative posture between the human and stroller are lacking, and the safety concern of the parents in the baby stroller scenario was not discussed sufficiently, especially for their human tracking systems. Furthermore, the proposed systems’ usability was not researched, and the effective hands-free control interfaces for a stroller are yet to be realized. Autonomous smart strollers offer greater freedom to parents while babysitting. The work in [3] proposes an autonomous motion control scheme using predictive control to safely and stably cope with difficult manual situations, such as sharp turns. The proposed method’s feasibility was validated in simulation with satisfactory results. However, the system can be affected by sensor noise and uncertain disturbances in the real-life scenario, which cause insecurity. Additionally, some parents feel uncomfortable being too far from the stroller or not being in control, which can lead to a feeling of insecurity. Therefore, considering practicality and addressing parents’ psychological concerns, we focus on the human-following or human-guided stroller in this work. We refer to the literature on human-following (or human-guided) robots due to the scarcity of research on human-following smart strollers, and due to the similarity in kinematics and technology between smart strollers and human-following robots.

### 1.1. Human Tracking Methods

Recognizing and localizing the target person is critical in a human-following robot system. Optical sensors are widely used to track a moving target [4,5]; however, their performance is affected by occlusion and light variation [6]. Laser range finders (LRFs) provide higher accuracy in terms of distance measurement, but they also suffer from occlusion. Optical sensors and LRFs are used in conjunction with algorithms for target recognition and tracking, which is not only computationally expensive but also can fail to track the target person or even reassign the target person under some circumstances [7]. Such risks make the optical sensors and LRFs unfavorable for the smart stroller application.

On the other hand, peer-to-peer localization systems using radio frequency benefit from identifying the target person carrying a tag and even under no line-of-sight (NLOS) conditions. Germa et al. built a human tracking system by fusing vision data and RFID data to enable the robot to follow a human in the crowd [8]. Liu et al. [9] proposed a human tracking method using passive ultra-high-frequency (UHF) RFID technology. RFID tags are cheap and small but their signal transmission can be easily hindered by physical obstructions and interference from other radio frequencies.

Geetha et al. [10] proposed a human-follow robot based on the WiFi RSSI-trilateration method by tracking the desired user with the phone. Conveniently, the user does not need to wear an extra sensor instead of carrying their phone in the human following robot situation. Other works [11,12] attempted to improve the localization accuracy of WiFi-based indoor positioning systems, and the proposed WiFi-based localization systems in the study [13] demonstrated an accuracy characterized by a one-meter spatial resolution in the indoor environment with obstacles. However, the signal strength of WiFi is affected by the obstacle, and localization performance is relatively inaccurate with meter-level accuracy.

The Bluetooth low energy (BLE)-based localization system is a popular solution in indoor positioning and object tracking [14,15]. It boasts advantages of being low cost and low power and is available in all recent smartphones. Reference [16] proposed a BLE-based human-following robot. However, like other RF technology, BLE localization can be affected by physical barriers and signal interference.

UWB sensors excel in localization precision, resistance to multipath interference [17], and minimize interference with other radio-frequency devices compared to other radio-frequency sensors above. Several works explored the possibility of UWB technology in the human-following robot field in recent years. Feng et al. proposed a person-tracking system only based on a UWB sensor for mobile robot navigation [18]. Hepp et al. mounted UWB radio transceivers on flying robots to build a target human-tracking environment [19], and Jin et al. proposed a robust human-tracking method by sensor fusion of UWB and LRFs [7].

The application scenario of a human-guided stroller extends to both indoor and outdoor environments. UWB sensors perform well in a line-of-sight (LOS) outdoor environment [20] and are minimally impacted by light exposure. Additionally, the UWB signal consists of various frequency components, which enhances its ability to penetrate obstacles due to its wide bandwidth [21], enabling it to perform well in the indoor environment with obstacles. It offers centimeter-level accuracy, is energy-efficient [17,22,23], and robustly enables tracking of the person even under NLOS conditions. In the smart stroller context, where it’s crucial to maintain proximity to the parent, a high degree of localization accuracy and reliable tracking is necessary. Additionally, major smartphone manufacturers now include UWB technology in their devices [24] to achieve precise localization and enhance device functionality, offering a good opportunity to leverage this technology for innovative applications, such as smart strollers.

Thus, the UWB sensors are suitably employed as a near-localization system for target tracking within a smart stroller framework. In a broader sense, we believe that the UWB sensor is a good candidate for accompanying robots to track the target human.

### 1.2. Human-Following Robot

Following behind the human, side-by-side with the human, and following in front of the human are the main categories of the human-following robot [25]. Following the target human from behind is investigated in the previous works [6]. In recent years, studies about following side-by-side and following in front are explored. Side-by-side walking is a human-like behavior and enables the robot to accompany the human, which is applied for social robots [26,27,28]. Following in front of a human is consistent with human behavioral patterns like pushing a cart and allows the human to check the robot at all times when the robot carries valuable items. We highlight the front-following studies since it is the same pose as our human-guided stroller.

Eui-Jung et al. [29] proposed a holonomic motion model to allow the robot to stay in front of the human. Cifuentes et al. proposed a natural following model using LRFs and IMU sensors to monitor movement in the gait cycle [30]. Yan et al. proposed a finite-time controller to control an omnidirectional cane robot to the desired pose using the user’s leg motion [31]. These works suffer from a common limitation; the lack of direct instructions from the user to the robot makes it difficult to handle situations where the users change their behavior suddenly, such as sharp turns.

To solve this issue, anticipatory control methods were proposed to enable the robot to predict the human’s next behavior [5,25]. Nikdel et al. proposed to estimate the user’s next action based on the current heading of the person and the position of the obstacle [32]. Dean et al. used the human head pose as an implicit indicator and predicted the human future pose based on the Bayesian technique and fused the predicted result with the other human pose-predicted result using Gaussian fusion. The proposed method was evaluated in a simulation with a comprehensive route design and achieved good results; however, it sometimes failed when passing constrained walk paths [33]. Using only anticipatory methods is not stable to keep the robot in the user’s FOV because of ambiguity in the user’s intention. Robots automatically attempt to reach a point in front of the person without direct human instructions in the above studies, which causes tracking failure in some cases.

### 1.3. Human-Guided Robot

Human-guided robots are controlled directly by the human operator, which allows the operator to interact with the robot explicitly. Various interactive strategies are designed to guide the robot in front of the operator. Leica et al. designed a physical interface to guide the robot by estimating the interaction force [34]. Young et. al. [35] developed a guiding system in which the user needs to approach the robot in a certain direction to rotate the robot in the opposite direction, analogous to pushing a cart virtually; however, only using this mode causes extra effort when turning a sharp curve happens often. Despite the progress achieved in the mentioned works, human–robot interaction (HRI) in human-guided robots has not been studied thoroughly. Smart walkers could also be categorized as human-guided robots since most of them are controlled by humans explicitly. For example, M. Scheidegger et al. proposed a method to control a smart walker’s rotation based on the face orientation of the user [36]. Smart walker users have direct physical contact with the walker, different from the scenario considered in this work in which the stroller precedes the user by a short distance. Therefore, the considered scenario requires additional supervision from the user over the robot, maintaining it in the field of view of the user. Overall, the explicit interaction methods provide unambiguous commands from the user to the robot, but it increases the user’s cognitive load since they have to explicitly control the robot at all times. In the case of a smart stroller, the parents are expected to remain aware of the baby’s condition and security as a priority; therefore, we consider that an explicit interaction approach is a suitable choice for the smart stroller regardless of its shortcomings.

Having investigated the methods of human tracking technologies and human-following and human-guided robots, in this work, we elected to use an ultra-wideband (UWB) localization system in the context of a human-guided stroller. We develop and evaluate a hands-free interface with human direct instruction for the smart baby stroller control utilizing the commercial UWB product. Potentially, the proposed system can leverage existing UWB-enabled smartphones to control the smart stroller, eliminating the need for the additional UWB tag carried by humans and ensuring seamless integration with everyday technology making it more user-friendly and practical.

The contributions of this paper are as follows:

1. This study presents information on the preferred positions of the stroller relative to the human based on questionnaires in a smart stroller scenario.
2. This study introduces a model of human-guided hands-free robot control based on the UWB localization system, in particular, the robot spatially precedes the human based on the preferred positions.
3. This study presents quantitative and qualitative evaluations of hands-free control of a smart stroller, compared to joy-stick control, and manual operation of the stroller. The results show the comparable performance between the hands-free interface and the joystick in controlling the stroller. The results of this work also show gender differences in the preference of a control interface, which is presented for the first time in the field of human-guided robot control, to the best of our knowledge.

In the following sections, we provide a comprehensive analysis of our study. Section 2 introduces the methodology, focusing on the preferred relative position between the human and the stroller, and the control methods employed. Section 3 offers an overview of the prototype developed for the study. In Section 4, we describe the experiments conducted, including Experiment 1: path-following test and Experiment 2: simulated real-life scenario, and the user evaluation. Section 5 and Section 6 present and discuss the results separately. Finally, Section 7 concludes the paper with a summary of key insights, implications, and limitations of our work, and potential directions for future research.

## 2. Methodology

### 2.1. Preferred Relative Positions between the Human and the Stroller

**Figure 1:**
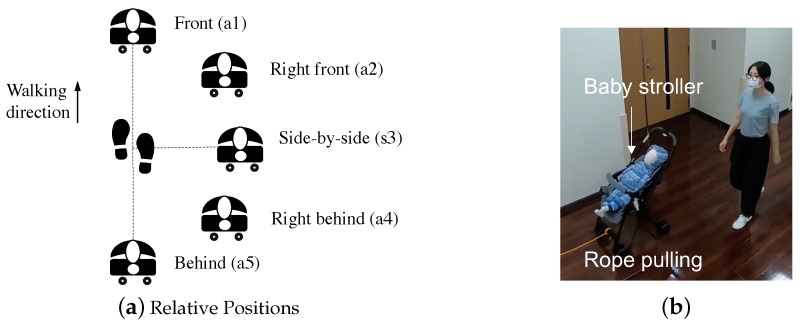
(**a**) Possible relative positions for the investigation. Five relative positions were considered: a1 (Front), a2 (Right front), a3 (Side-by-side), a4 (Right behind), and a5 (Behind). (**b**) Sample of the video for preferred position investigation: Right front position as an example, a rope is used to pull a commercialized normal stroller (with a baby doll inside) at a constant speed to imitate the human-following behavior.

**Figure 2:**
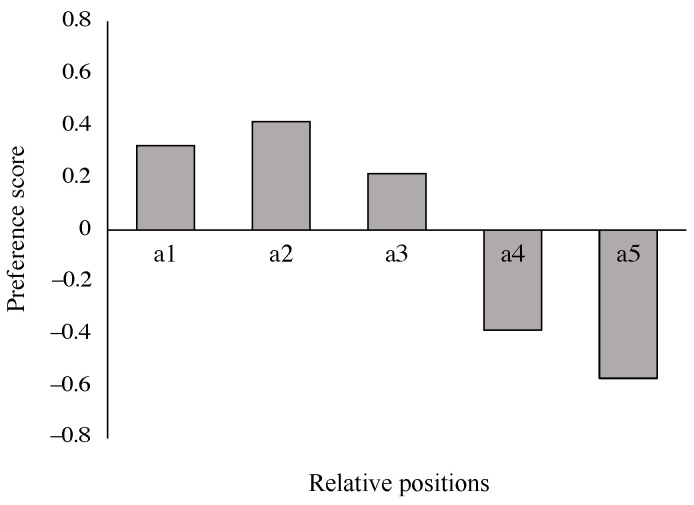
Results of the participants’ preferred position relative to the stroller. The X-axis represents human-stroller relative positions: a1: Front, a2: Right front, a3: Side-by-side, a4: Right behind, a5: Behind (Figure 1a). The Y-axis shows the preference score of all participants for each relative position.

In a human-following or a human-guided locomotion task, the goal of the control algorithm is to ensure the convergence of the robot to the target pose or position. The criteria for selecting a target pose while following is unspecified but with task-specific reasoning [37]. We investigated the target position in the case of a hands-free smart stroller. We assumed five possible relative positions, Front, Right-front, Side-by-side, Right-behind, and Behind, as noted as a1∼a5 separately in Figure 1a. A questionnaire survey was conducted to investigate the preferred relative positions among these five cases. We created five videos to simulate the different relative following positions in motion using a traditional stroller pulled by a rope. Figure 1b illustrates an image from one of the five video simulations of the stroller following the person with the relative position, right front (a2), others are similar to this one, but with different relative positions. We divided the five videos into 10 pairs for comparison. In each pair, the first following type is noted as type 1 and the second as type 2. Participants were asked to watch one group of videos and answer the question, “Following type 1 is preferred over type 2” with five answer choices from strongly disagree (−1) to strongly agree (1). Thirteen participants (aged between 28 to 40 years) joined this survey, all of them are parents who are using a stroller or have used a stroller in recent years. The average preference scores for the five candidate relative positions for all participants are shown in Figure 2. Positive numbers in the score indicate a positive attitude toward the relative pose, and conversely, negative numbers indicate a less favorable attitude toward the relative pose. The results were analyzed using Sheffe’s Paired Comparison (Nakaya Variation). The results indicated that all participants exhibited a strong preference for the stroller positions: Front, Front-right, and Side-by-side, and showed a marked disfavor for the positions Behind-right and Behind. We hypothesize that these results are related to a preference for the stroller to move in the FOV of the human, giving the parent a better sense of security of the child in the stroller. Notably, positions Front (a1) and Front-right (a2) received the highest preference scores, suggesting general favoritism towards this positioning. Based on these findings, we have selected the Front as the following position for the controller design and development of the proposed system.

### 2.2. Human State Estimation

A UWB polar frame *U* is attached to the UWB anchor. The robot’s polar frame *R* is attached to the center of the robot. The UWB tag is fixed to the user’s waist. The human position $H_{U}\left(d^{*},\alpha^{*}\right)$ in the UWB frame is assumed to coincide with the tag, which can be transformed into the robot frame as $H_{R}\left(d,\alpha \right)$, derived by the following formula:

(1) $$\left\{\begin{array}{c} d=\sqrt{{\left(d^{*}\cdot \sin\alpha^{*}\right)}^{2}+{\left(d^{*}\cdot \cos\alpha^{*}-L\right)}^{2}}\\ \alpha =\mathrm{atan}2\left(d^{*}\cdot \sin\alpha^{*},d^{*}\cdot \cos\alpha^{*}-L\right) \end{array}\right.,$$

where *L* is the length of UR, which is a constant and equal to 0.75 m, $d^{*}$ denotes the length of HU, $\alpha^{*}$ denotes the angle from UR to UH. *d* denotes the length of HR, α denotes the angle from the robot’s centerline to RH. Human locomotion is assumed as a holonomic model [38] in this work and its state is denoted as:

(2) $$X_{H}={\left[\begin{array}{cccc} x_{H} & y_{H} & \dot{x_{H}} & \dot{y_{H}} \end{array}\right]}^{⊤}$$

where the terms $x_{H}$ and $y_{H}$ represent the coordinates of a human in the global frame, while $\dot{x_{H}}$ and $\dot{y_{H}}$ denote the human’s walking speeds in the x and y directions of the global frame, respectively. Within each sampling interval ΔT, human motion while walking can be regarded as uniform motion. The pose of the robot $R_{O}\left(x_{r},y_{r},\theta_{r}\right)$ in the world frame is obtained through visual odometry. Combining $R_{O}$ and $H_{R}$, we could obtain the position of the human in the world frame. Then, we fuse it with the human locomotion model to attenuate the noise by utilizing an extended Kalman filter (EKF). The estimated human state is denoted as $\hat{X_{H}}$($\hat{x_{H}},\hat{y_{H}},\hat{\dot{x_{H}}},\hat{\dot{y_{H}}})$.

### 2.3. Control Method

**Figure 3:**
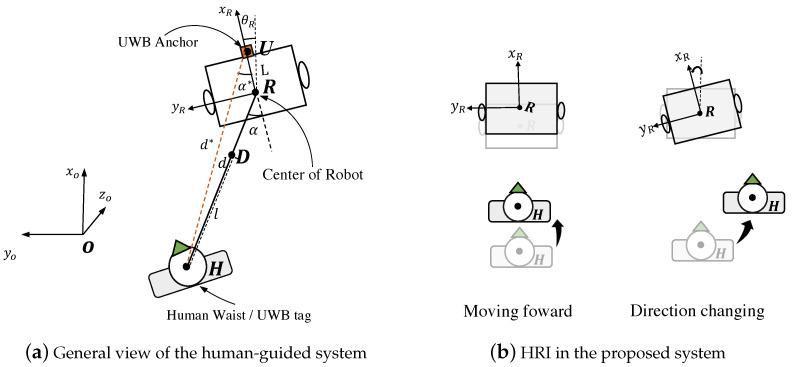
(**a**) Top view illustration of the human-guided system. (**b**) The proposed human-guided system: the human adjusts the relative distance to virtually push the robot moving forward and virtually rotates the robot direction with distance-shift.

The control method and human–robot interaction are described in this section. We implemented a hands-free interface method to allow the parents to control the stroller’s movement simply using their body movement. The control method is shown in Figure 3a, *D* denotes the destination of the stroller, which is located in the line of the HR with a length of *l* from *H*. The distance error d−l and angle error α are used to control the stroller. The stroller continuously attempts to move directly in front of the tracked person’s current position and reach point *D* to maintain the distance *l* from the user. The user needs to perform distance-shift behavior to rotate the stroller towards the opposite direction like virtually pushing the stroller as shown in Figure 3b. Based on the estimated human position $\hat{X_{h}}$($\hat{x_{h}},\hat{y_{h}})$ and stroller state $\left(x_{r},y_{r},\theta_{r}\right)$, the estimated length of HR $\hat{d}$ and angle from the stroller’s centerline to RH $\hat{\alpha }$ could be calculated by

(3) $$\left\{\begin{array}{c} d_{t}=\sqrt{{\left(x_{r}-{\hat{x}}_{h}\right)}^{2}+{\left(y_{r}-{\hat{y}}_{h}\right)}^{2}}\\ \alpha_{t}=\mathrm{atan}2\left(y_{r}-{\hat{y}}_{h},x_{r}-{\hat{x}}_{h}\right)-\theta_{r}\\ \hat{d}=\sqrt{{\left(d_{t}\cdot \sin\alpha_{t}\right)}^{2}+{\left(d_{t}\cdot \cos\alpha_{t}-L\right)}^{2}}\\ \hat{\alpha }=\mathrm{atan}2\left(d_{t}\cdot \sin\alpha_{t},d_{t}\cdot \cos\alpha_{t}-L\right) \end{array}\right.$$

The linear velocity *v* and angular velocity *w* are controlled by Equations (4) and (5) separately.

(4) $$\left\{\begin{array}{cc} v=k_{p}^{d}\cdot e_{d}+k_{d}^{d}\cdot \dot{e_{d}} & |e_{d}|>{th}_{d}\\ v=0 & |e_{d}|\leq \;{th}_{d} \end{array}\right.,$$

(5) $$\left\{\begin{array}{cc} w=k_{p}^{\alpha }\cdot e_{\alpha }+k_{d}^{\alpha }\cdot \dot{e_{\alpha }} & |e_{\alpha }|>{th}_{\alpha }\\ w=0 & |e_{\alpha }|\leq \;{th}_{\alpha } \end{array}\right.,$$

where $e_{d}=\hat{d}-l$, $e_{\alpha }=\hat{\alpha }$, [$-{th}_{d}$, ${th}_{d}$], and [$-{th}_{\alpha }$, ${th}_{\alpha }$] are the velocity dead zone, which is used to reduce the stroller’s sensitivity when the human is in still standing in place or making slight movements, such as turning one’s body to talk to someone nearby.

## 3. Prototype Overview

**Figure 4:**
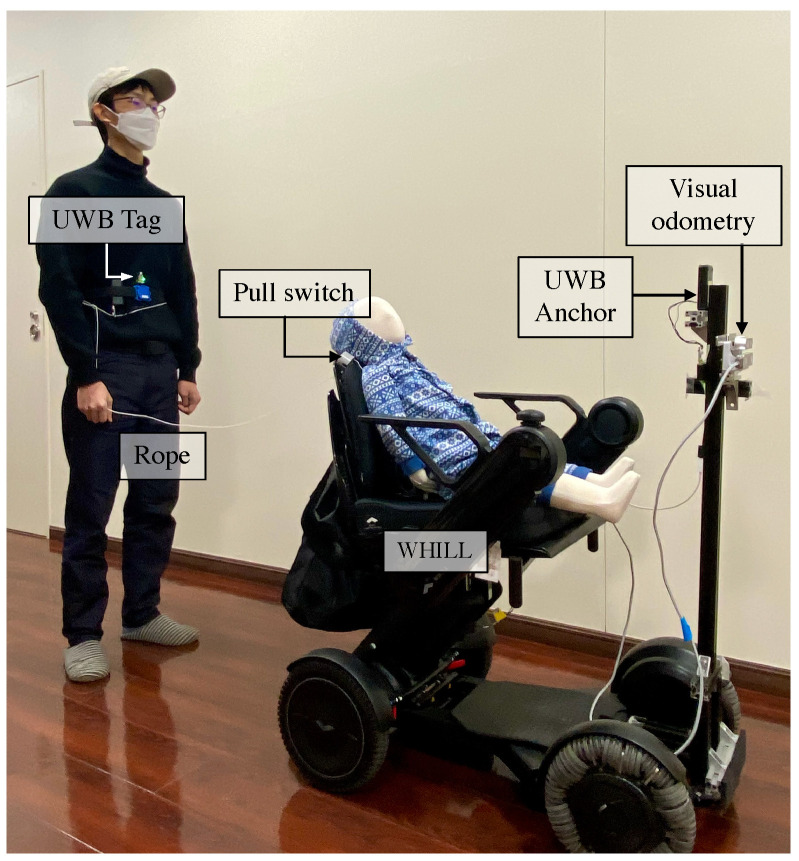
System Overview. Comprehensive design and components of the proposed system. The system is implemented on a commercial electric wheelchair, equipped with an ultra-wideband (UWB) anchor positioned at the front. The human guides the wheelchair while wearing a UWB tag on the waist. Additionally, a leash-type switch connects the user’s waist to the wheelchair, increasing the sense of security and enhancing the capacity to handle emergencies.

**Figure 5:**
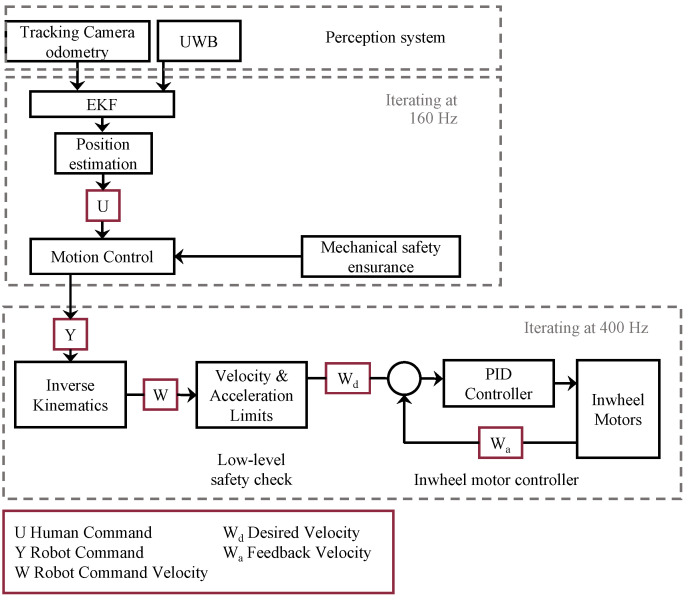
Overall control architecture for human-guided robotics system.

To test the performance of the proposed system, we implemented a prototype on the wheelchair WHILL (Model C2, WHILL Inc., Tokyo, Japan), which is a non-holonomic mobile platform. The details of the components of the prototype are summarized in Table 1. The UWB sensor we selected is LinkTrack AOA (SZ Nooploop Technology Co., Ltd., Shenzhen, China). We devised a preliminary test to measure the performance of the UWB sensor in a short-distance (3 m) indoor environment. In the preliminary test, we collected data from the measurement points in a semi-circular pattern with angular positions at −75, −60, −30, and 0 degrees relative to the anchor. These points are located at radial distances of 0.5, 1.0, 1.5, and 2 m from the anchor. Additionally, there are two measurement points positioned on the vertical axis (0 degrees) at distances of 2.5 m and 3 m. The sampling rate of the UWB sensor we used was set as 100 HZ, the measurement time was 5 s at each point. Ten trials were conducted for each point to obtain a reliable measurement result. The outcome revealed that distance measurement and angle measurement achieved stable results with smaller errors (distance error = 15 cm, angle error = 5 degrees) at the angle of 0 degrees and distance of 2 m. Based on the outcome of the preferred relative positions and the UWB measurement errors, we mounted the UWB anchor at the front of the robot and set the following distance *l* to 0.85 m so that the distance between the anchor and tag was close to 2 m. We fixed the UWB tag on the target human’s waist as shown in Figure 4. A visual odometry sensor (Realsense T265, Intel Corp., Santa Clara, California, USA) was mounted on the front of the WHILL. In addition to functional safety, such as the robust perception method, a rope physically connects the user and the stroller as mechanical insurance. A pull switch is mounted on the stroller and can be operated through the rope, the user could pull the switch to interrupt the automatic control of the stroller when intervention is needed. The stroller could resume moving when the user pulls the switch again. The pull switch is located on the backside of the wheelchair. A Jetson Nano (Nvidia Corp., Santa Clara, California, USA) was used to implement the control logic. The modular design of the robot operation system (ROS) enhances system development flexibility and scalability by enabling independent development, testing, and deployment of components. Supported by a vast community, ROS offers access to extensive libraries and essential tools like RViz and Gazebo, which accelerates development and addresses complex challenges. Additionally, ROS facilitates interoperability by easily integrating diverse hardware and software components through robust inter-node communication capabilities, accommodating a wide range of sensors and actuators. Therefore, we implemented software using ROS running on Ubuntu 20.04. The overall control architecture is presented in Figure 5.

## 4. Experiments

We conducted two experiments to evaluate the proposed system. The first experiment evaluates if the human could guide the robot to follow a trajectory. The second experiment aims to observe the general controllability of the whole system in a simulated daily life scenario. A total of 14 participants joined the experiments, seven males (29.57±4.28 years) and seven females (38.42±13.83 years). Five of the participants are parents and two of them currently use a baby stroller. A virtual joystick (smartphone app) was used for comparison. For both experiments, participants repeated the same task three times using the human-guided system, the virtual joystick, and manual operation in a randomized order. The general procedure was: 1. explanation of the human-guiding system/virtual joystick; 2. training session (around 10 min); 3. measurement trials.

### 4.1. Experiment 1: Path-Following Test

**Figure 6:**
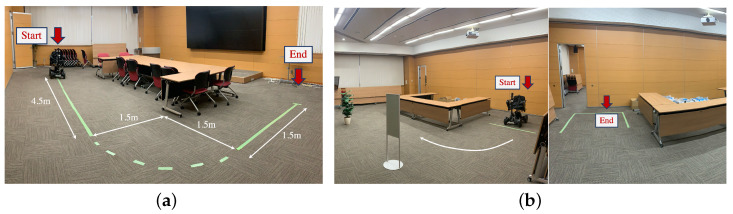
Experiment setup. (**a**,**b**) The setup of the precision control test and simulated real-life experiment. For (**a**), the users were asked to follow the indicator lines posted on the ground as precise as possible; for (**b**), the path was used as a direction guidance only.

The setup of Experiment 1 is shown in Figure 6a. The trajectory is sequentially composed of a 4.5 m straight line, a quarter circle (r = 1.5), and a 1.5 m straight line around a corner. Participants were instructed to use the three interfaces to operate the stroller and follow the lines on the floor. We utilized the Procrustes Distance to present the dissimilarity between the experimental robot and ground truth trajectories.

- Procrustes Distance The Procrustes distance is a measure of dissimilarity between shapes based on Procrustes analysis. The Procrustes function finds the best shape-preserving Euclidean transformation between two shapes. In this work, we compare the two trajectories Traj1 and Traj2 using the Procrustes analysis, the trajectories would be optimally superimposed, including translating, rotating, and uniformly scaling, to minimize the Procrustes Distance between transformed metrics m1 and m2. The Procrustes Distance (PD) is calculated by
  (6) $$PD\left(m1,m2\right)=\sqrt{\sum_{j=0}^{n}\sum_{i=0}^{k}{\left(x_{ij}^{m1}-x_{ij}^{m2}\right)}^{2}}.$$
  Here, $x_{ij}^{m1}$, $x_{ij}^{m2}$ are the coordinates of the *i*-th point in shapes m1 and m2, separately; n is the number of points on the trajectory; and *k* is the spatial dimensions. The Procrustes distances between the experimental robot and ground truth trajectories are calculated using the Python library [39]. The returned numeric scalar is within [0, 1], with higher values representing less similarity.

### 4.2. Experiment 2: Simulated Real-Life Scenario

Experiment 2 was conducted in an indoor environment with static obstacles; the experiment condition is depicted in Figure 6b. No other humans were in the vicinity of the experiment except the experimenters. The task was to start guiding the stroller with three interfaces from the right area to the left carrying a shopping bag (about 2 kg). During the process, the participants were free to choose the trajectory as to their preference without any special instructions. The completion time for operating the stroller using three different interfaces was recorded.

### 4.3. User Evaluation

After finishing all tasks, questionnaires were distributed to the participants to investigate their evaluation of the proposed human-guided system (hands-free interface), virtual joystick, and manual operation. Each questionnaire contained three parts, the system usability scale (SUS) [40], NASA-TLX [41], and an additional questionnaire containing four items as follows: 1. I found that it was smooth to control the robot. 2. I found the skills of the system well-suited for the task. 3. I felt that interacting with the robot was natural. 4. I found that using the system was safe. The answers were selected from strongly disagree (1) to strongly agree (5). The participants were instructed to answer the questionnaires based on their experience on two tasks. After filling in the questionnaire, we listened to the free feedback from the participants as well.

## 5. Results and Analysis

The results of the two experiments are described in this section. We assessed the human-guided system through path dissimilarity in Experiment 1, the completion time of Experiment 2, and the user evaluation after all experiments, compared to the joystick and manual operation. The results were categorized into three groups, all participants group, the male group, and the female group to observe the gender difference.

### 5.1. Path-Following Results: Experiment 1

**Figure 7:**
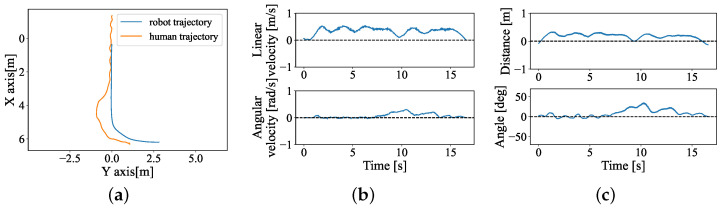
One example result of Experiment 1 with the human-guided system. (**a**) Trajectory of the robot and human. The robot starts from (0, 0). (**b**) Robot real linear velocity (top) and angular velocity (bottom). (**c**) Results of $e_{d}$ and $e_{\alpha }$.

**Figure 8:**
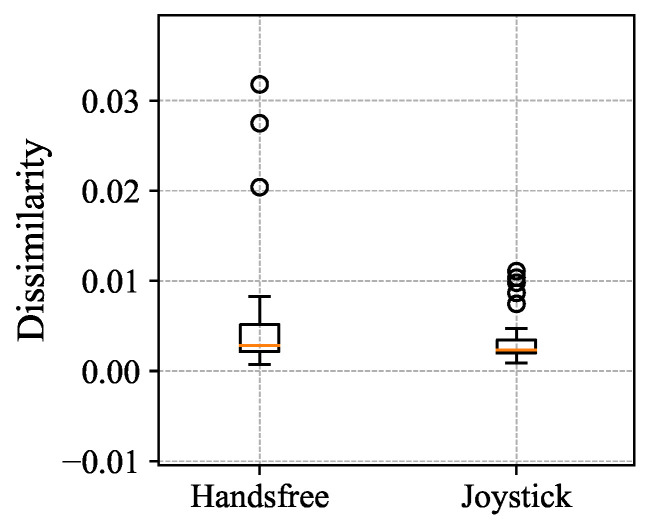
Results of dissimilarity with the human-guided system (handsfree) and with the virtual joystick.

All participants finished all of the tasks successfully. One example trial is shown in Figure 7, trajectories of the participants and stroller, the linear and angular velocities are shown in (a) and (b) in Figure 7. The data source was directly obtained from visual odometry and the UWB sensor. The estimated distance error and estimated angle error are shown in (c); both errors converge to zero near the end of each recording. Figure 8 illustrated the path dissimilarity results of the hands-free interface and joystick for all participants. The dissimilarity of the human-guided system is 0.0126±0.0385, which is higher than 0.0008±0.031 of the joystick. Furthermore, there is no significant difference between the two interfaces from the results of the Wilcoxon Signed-Rank Test, indicating that the position control precision of the human-guided system is close to that of the joystick.

### 5.2. Completion Time Results: Experiment 2

**Figure 9:**
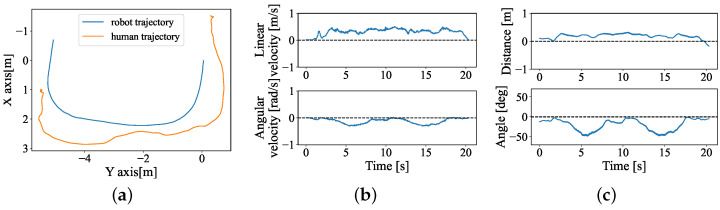
One example result of Experiment 2 with the human-guided system. (**a**) Trajectory of the robot and human. The robot starts from (0,0). (**b**) Robot real linear velocity and angular velocity. (**c**) Results of $e_{d}$ and $e_{\alpha }$.

**Figure 10:**
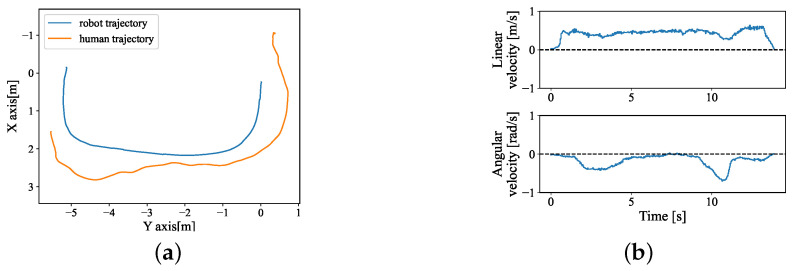
One example of Experiment 2 with joystick control. (**a**) Trajectory of the robot and human. The robot starts from (0,0). (**b**) Robot real linear velocity and angular velocity.

**Figure 11:**
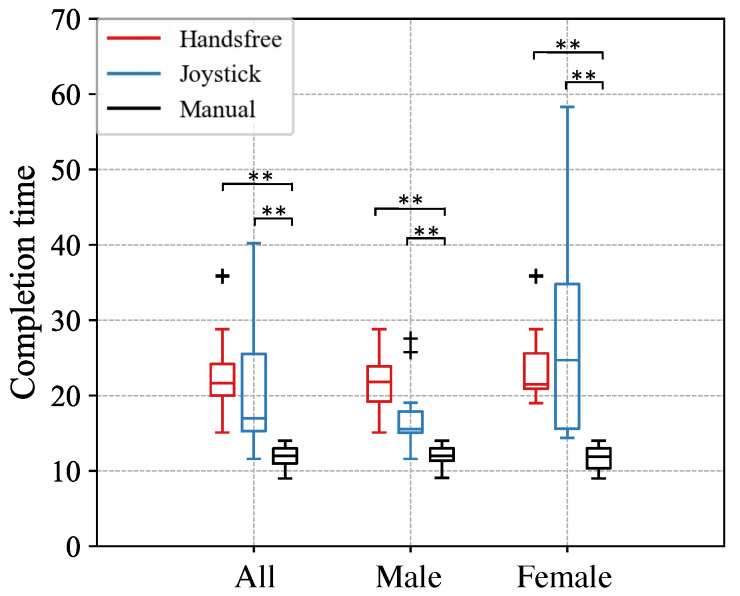
Results of completion time of Experiment 2. ** denotes a significant difference at the level *p* < 0.003 (Bonferroni correction).

All participants could successfully guide the robot from the start area to the end area using three interfaces. One example of the trajectory and velocity result using the human-guided interface and joystick interface is shown separately in Figure 9 and Figure 10. The completion time of the three interfaces is illustrated in Figure 11. Based on the results of the Wilcoxon Signed-Rank Test with Bonferroni correction, we observed that the hands-free interface and joystick significantly cost the participants more time to finish this task across all participants and consistently for the male or female category. Across all categories, there was no significant difference between the hands-free interface and the joystick, with subjects taking, on average, about 2 s longer with the hands-free interface than with the joystick (hands-free = 23.01 s, joystick = 21.85 s). Males and females spent a similar average time operating the human-guided interfaces. Still, the male group spent less time finishing the task than the female group when using the joystick with a small standard deviation.

### 5.3. User Evaluation Results: Experiments 1 and  2

**Figure 12:**
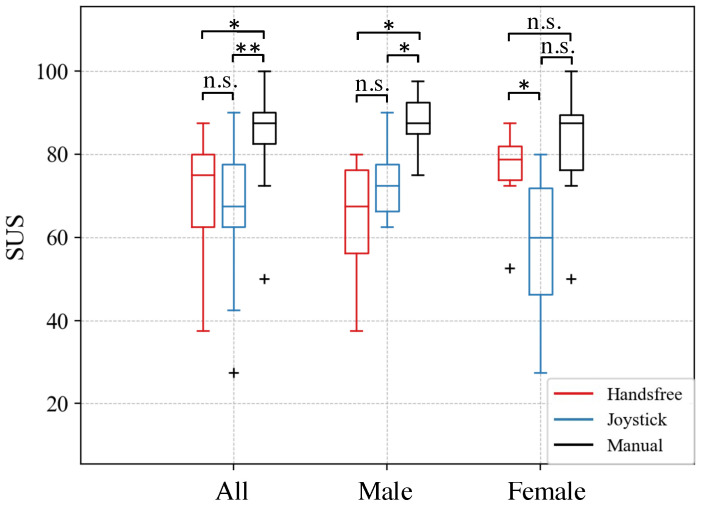
Results of the system usability scores of the proposed hands-free interface, joystick, and manual operation. ** and * denotes a significant difference at the level *p* < 0.003 (Bonferroni correction) and *p* < 0.017 (Bonferroni correction) separately. n.s. denotes no significant difference.

The system usability scale results for all subjects, male subjects and female subjects for three interfaces are shown in Figure 12. The average and standard deviation of the SUS scores are summarized in Table 2. Wilcoxon Signed-Rank Test (Bonferroni correction) was conducted to compare the usability scale across the three interfaces. The analysis demonstrated that, for all participants, the manual operation had a higher score than the other two interfaces, the hands-free interface, and the joystick exhibited significant differences compared to manual operation. No significant difference was observed between the hands-free interface and the joystick. For male subjects, the statistical results were consistent. However, for female subjects, the findings were the opposite. A significant difference was observed in the hands-free interface scores higher than the joystick. No significant differences were found when comparing the hands-free interface to manual operation, or the joystick to manual operation.

**Figure 13:**
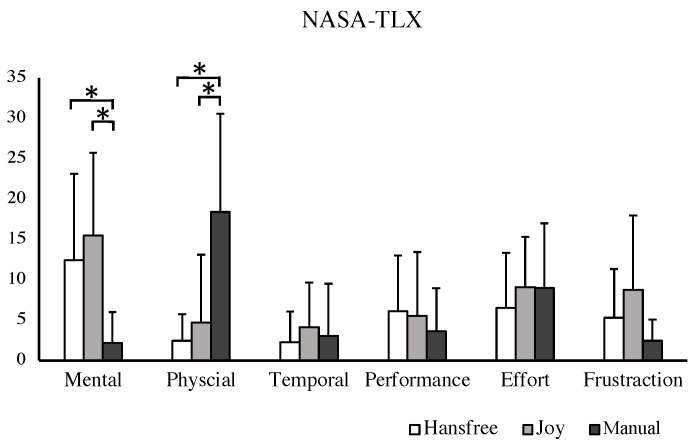
Results of the Nasa-TLX for all subjects. * denotes a significant difference at the level *p* < 0.017 (Bonferroni correction).

Scores for the NASA Task Load Index dimension for all participants are shown in Figure 13. Based on the results of the Wilcoxon Signed-Rank Test with Bonferroni correction, differences in task load are primarily manifested in terms of mental demand and physical demand across three interfaces. Hands-free and joystick showed higher mental demand than manual operation with significant differences observed. However, they presented significantly lower physical demand compared to manual operation for all participants.

**Figure 14:**
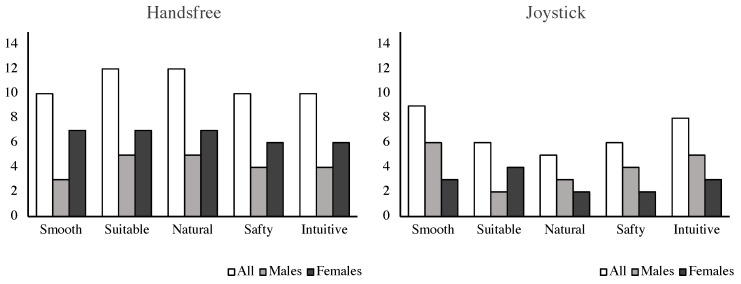
Results of the additional questionnaire, smoothness, suitness. naturalness, safety, and intuition.

The scores of the additional questionnaire are illustrated in Figure 14. For all participants and female subjects, the hands-free interface appeared to perform better in smoothness, suitableness, naturalness, safety, and intuition than the joystick. While male subjects reported that the proposed method performed better in task suitableness, and naturalness but worse in intuition and smoothness compared to the joystick, the two interfaces had the same level of safety.

## 6. Discussion

The discussion begins with an analysis of the controllability of the proposed system, comparing it to the virtual joystick’s performance. This is followed by a discussion of the system’s usability across three stroller operation methods: the human-guided system, joystick control, and manual operation. Lastly, the discussion addresses the system’s task load and safety insurance.

### 6.1. Controllability

**Figure 15:**
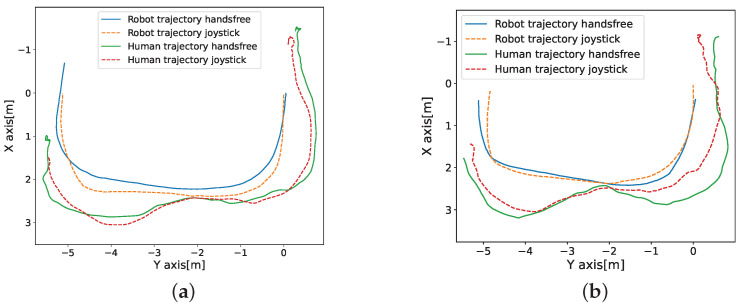
Two examples of human/robot trajectory under the hands-free control and joystick control with different objects. (**a**) Example one (**b**) Example two.

From the results of the first experiment, we found that both the proposed hands-free method and the virtual joystick achieved low path dissimilarity scores when comparing the target trajectory to the achieved trajectory. The joystick produced a better score with no significant difference observed, which shows that the two interfaces have similar control precision for the target trajectory. Comparing the robot/human trajectory under the human-guided interface and joystick in Figure 15, it can be observed that the robot trajectories under the two interfaces are similar. Due to the distance-shift steering control method, the participants chose a larger turning radius when steering the stroller using the hands-free interface than the joystick. Also, the participants did not consistently stay directly behind the stroller while maneuvering using the joystick, especially when walking in the opposite direction, they wanted to turn during the steering process. This distance-shift behavior is similar to that in the proposed system, demonstrating that the operation method in the proposed system was intuitive to the participants. This result is consistent with our findings in the preference following the relative position survey (Section 2.1) that it’s acceptable for humans to walk with the smart stroller in a proximity front area. This phenomenon may be due to natural human behavior or the need for humans to maneuver a human-guide mobile device around a corner to gain a larger viewing angle through distance adjustment. Still, in the interview, some of the male participants reported that the joystick is more sensitive and easy to direct compared to the human-guided system. Males gave the joystick a higher score on the smoothness item in the additional questionnaire. On the contrary, the females gave the opposite score. This observation is consistent with the literature on sex differences in visuomotor tracking, showing an advantage for males in eye-hand coordination, such as in using a joystick [42].

### 6.2. System Usability

As the novel operation methods, two electrical-assisted interfaces, the proposed interface and joystick, have a lower usability scale than the traditional manual operation across all participants in the stroller scenario. The proposed method seemed no less usable than the joystick, a standard interface with a higher median value usability scale. Furthermore, it has sufficient performance in the completion time of the second task even though the joystick has a faster maximum speed. Gender differences should be considered, as the findings revealed that females achieved significantly higher scores with the hands-free interface than the joystick, with no significant difference from manual operation. In contrast, males did not exhibit significant differences, and their median score for the hands-free interface was lower and significantly different from manual operation. This may be due to the difference in performance between males and females on joystick usage. Similarly, studies [43,44] reported that men typically outperform women in tasks like video games and VR driving simulations using a joystick. That might be because males and females have different performances in visuomotor tracking. Mathew et al. [42] noted that males show a clear advantage in hand tracking accuracy during a visuo-oculo-manual motor task and women exhibit a larger temporal lag between hand motion and target motion. The reasons for this phenomenon were further investigated by Cherney, Isabelle D [45], and Nenna et al. [46]. They hypothesize that the gap in joystick performance is primarily due to varying levels of experience and practice with joystick-based activities, rather than gender itself being a determining factor. In our experiment, most of the female subjects stated that they have less joystick usage experience yet most of the male participants have experience with the joystick. They also mentioned that the unfamiliarity with joystick operations caused them to pay more attention when operating the joystick while maneuvering the stroller, making it harder to check the surroundings. Additionally, female subjects reported that the proposed interface was intuitive to use, and rated it higher on all aspects in the additional questionnaire compared to the joystick. This may explain their preference for the hands-free interface. The proposed interface is thereby indicated to be user-friendly and easy to learn even for those with little to no prior experience with joystick-based activities, such as, female and some elderly users.

The experiment verified the feasibility and effectiveness of the proposed system in a simplified indoor environment with static obstacles. However, the outdoor condition is more complex with bumpy roads, and slopes we have not tested this time. These factors may affect system performance and user experience, raising challenges to control stability and accuracy. The UWB transmitter and receiver may not remain on the same plane due to the stroller bouncing on bumpy roads and the stroller not being on the same plane as the person while moving uphill or downhill. This could lead to a decrease in the UWB sensor’s accuracy in localization, or it could cause minor angular differences even when the person does not intend to steer the stroller to turn. Localization errors will directly impact the vehicle’s speed commands; however, the impact can be mitigated to a certain extent by the EKF. Unexpected distance shifts can be offset due to the setting of the velocity dead zone to ensure control stability. We infer our system can be less affected by such road conditions to a certain extent. Furthermore, confined spaces such as the elevator require a system that provides more accurate operation. In these scenarios, switching to manual operation is the best option for controllability and safety.

### 6.3. Task Load

In terms of the task load, in the task of the simulated real-life scenario, we designed the experiment condition of holding one shopping bag while operating the stroller with three interfaces which is a simulated real-life scenario with a higher physical load for the parent. The results of NASA TLX demonstrated that the electrically assisted stroller significantly increases the mental demand in comparison to manual operation contrary to showing a lower physical demand. A greater cognitive effort is justifiable because of unfamiliarity, but it is anticipated to lessen with continued use. The lower average score in physical and mental demand indicated that the proposed interface has an advantage over joystick-based interfaces when one of the user’s hands is occupied. This advantage would be even more pronounced when both of the user’s hands are occupied. This is corroborated by similar feedback from the participants that the proposed method will be helpful when two hands are occupied while operating the stroller, especially for the participant who is a parent currently using a baby stroller.

### 6.4. Safety Insurance

The safety of the system is guaranteed in three aspects. First, the tracking method is based on UWB, which ensures functional safety. UWB could keep tracking the target person with strong anti-interference capability, even when there is obstruction. Although the precision is not perfect, with filtering, it is suitable for the target application scenario. Second, the control algorithm requires the stroller always to maintain a proximate distance from their parents (about one meter), which guarantees that the stroller is easy for the patient to reach the baby. Third, the safety mechanism, the rope and pull switch, not only prevents other people from interfering between the stroller and the user but also gives the user authority to stop the stroller whenever danger is predicted. After the experiments, some participants reported that the safety insurance mechanism is very important for their mental relief.

## 7. Conclusions and Future Work

In this work, we designed a hands-free human-guided smart stroller. We investigated the users’ preference for the relative following position and proposed a human-guided control system using the UWB localization system. The interaction in the proposed system allows the user to operate the smart stroller intuitively and ensures that the stroller remains within an appropriate range of the user. An experiment was conducted indoors to assess the proposed interface’s usability and effectiveness compared to a virtual joystick and manual operation. The results showed that the proposed system achieved an SUS score comparable to the joystick in terms of usability, yet inferior to manual operation. Notably, among the female participants the SUS score surpassed the joystick in usability and was comparable to the manual operation. Moreover, the proposed interface significantly reduced the physical demand compared to the manual operation on the occasion when the user’s hands were occupied. The proposed system is designed for a baby stroller; however, it can also be generalized to wheelchairs for caregiving scenarios.

The proposed system has the following limitations. The design principle of the proposed system focuses on the ease of implementing a UWB localization-based smart stroller, meeting the basic requirements for stroller control. We assume that the usage scenario for human-guided smart strollers is in less crowded, open areas such as parks. Therefore, we did not place excessive emphasis on collision avoidance. This will decrease the risk of collision when the user drives the stroller. An improved controller with a simple collision avoidance function will be considered in future works. Additionally, only a subset of the participants in the current evaluation experiment were actual parents. Considering that parents and non-parents might exhibit different capabilities and responsibilities in using strollers, the results may not fully reflect parental behavior. Consequently, future research will specifically target parents to more effectively assess the proposed system.

## Figures and Tables

**Table 1:** Components of the prototype.

Component	Details
Platform	Model C2 (WHILL)
UWB sensor	LinkTrack AOA (Nooploop)
Visual odometry	T265 (Realsense)
Microcomputer	Jetson Nano (Nvidia Corporation)
Canopy switch	WS5201HP (Panasonic)

**Table 2:** SUS score.

	Human-Guided (Hands-Free)	Joystick	Manual
All	69.43 (14.79)	65.96 (16.47)	84.81 (13.17)
Male	64.29 (15.66)	73.21 (9.43)	87.85 (7.96)
Female	75.42 (12.29)	57.50 (19.81)	81.25 (17.66)

## Data Availability

Data are contained within the article.

## Abbreviations

The following abbreviations are used in this manuscript:

UWB	Ultra-wide-band
AOA	Angle of arrival
LRF	Laser range finder
IMU	Inertial measurement units
FOV	Field of view
RFID	Radio frequency identification
BLE	Bluetooth low energy
LOS	Line of sight
NLOS	No line of sight
HRI	Human–robot interaction
EKF	Extended Kalman filter
SUS	System usability scale
NASA-TLX	NASA Task Load Index

## References

[1] Smartbe Intelligent Stroller. https://www.indiegogo.com/projects/smartbe-intelligent-stroller#/.

[2] Raihan M.J., Hasan M.T., Nahid A.A. (2022). Smart human following baby stroller using computer vision. Khulna Univ. Stud..

[3] Zhang C., He Z., He X., Shen W., Dong L. (2022). The modularization design and autonomous motion control of a new baby stroller. Front. Hum. Neurosci..

[4] Wang M., Su D., Shi L., Liu Y., Miro J.V. Real-time 3D human tracking for mobile robots with multisensors. Proceedings of the 2017 IEEE International Conference on Robotics and Automation (ICRA).

[5] Mi W., Wang X., Ren P., Hou C. A system for an anticipative front human following robot. Proceedings of the International Conference on Artificial Intelligence and Robotics and the International Conference on Automation, Control and Robotics Engineering.

[6] Islam M.J., Hong J., Sattar J. (2019). Person-following by autonomous robots: A categorical overview. Int. J. Robot. Res..

[7] Jin D., Fang Z., Zeng J. (2020). A robust autonomous following method for mobile robots in dynamic environments. IEEE Access.

[8] Germa T., Lerasle F., Ouadah N., Cadenat V. (2010). Vision and RFID data fusion for tracking people in crowds by a mobile robot. Comput. Vis. Image Underst..

[9] Liu R., Huskić G., Zell A. (2015). On tracking dynamic objects with long range passive UHF RFID using a mobile robot. Int. J. Distrib. Sens. Netw..

[10] Geetha V., Salvi S., Saini G., Yadav N., Singh Tomar R.P. (2021). Follow me: A human following robot using wi-fi received signal strength indicator. Proceedings of the ICT Systems and Sustainability: Proceedings of ICT4SD 2020.

[11] Altaf Khattak S.B., Fawad, Nasralla M.M., Esmail M.A., Mostafa H., Jia M. (2022). WLAN RSS-based fingerprinting for indoor localization: A machine learning inspired bag-of-features approach. Sensors.

[12] Khattak S.B.A., Jia M., Marey M., Nasralla M.M., Guo Q., Gu X. (2022). A novel single anchor localization method for wireless sensors in 5G satellite-terrestrial network. Alex. Eng. J..

[13] Magsino E.R., Sim J.K., Tagabuhin R.R., Tirados J.J.S. Indoor Localization of a Multi-story Residential Household using Multiple WiFi Signals. Proceedings of the 2021 International Conference on Innovation and Intelligence for Informatics, Computing, and Technologies (3ICT).

[14] Bai L., Ciravegna F., Bond R., Mulvenna M. (2020). A low cost indoor positioning system using bluetooth low energy. IEEE Access.

[15] Yu Y., Zhang Y., Chen L., Chen R. (2023). Intelligent fusion structure for Wi-Fi/BLE/QR/MEMS sensor-based indoor localization. Remote Sens..

[16] Pradeep B.V., Rahul E., Bhavani R.R. Follow me robot using bluetooth-based position estimation. Proceedings of the 2017 International Conference on Advances in Computing, Communications and Informatics (ICACCI).

[17] Kunhoth J., Karkar A., Al-Maadeed S., Al-Ali A. (2020). Indoor positioning and wayfinding systems: A survey. Hum.-Centric Comput. Inf. Sci..

[18] Feng T., Yu Y., Wu L., Bai Y., Xiao Z., Lu Z. (2018). A human-tracking robot using ultra wideband technology. IEEE Access.

[19] Hepp B., Nägeli T., Hilliges O. Omni-directional person tracking on a flying robot using occlusion-robust ultra-wideband signals. Proceedings of the 2016 IEEE/RSJ International Conference on Intelligent Robots and Systems (IROS).

[20] Jiménez A.R., Seco F. Comparing Decawave and Bespoon UWB location systems: Indoor/outdoor performance analysis. Proceedings of the 2016 International Conference on Indoor Positioning and Indoor Navigation (IPIN).

[21] Elsanhoury M., Mäkelä P., Koljonen J., Välisuo P., Shamsuzzoha A., Mantere T., Elmusrati M., Kuusniemi H. (2022). Precision positioning for smart logistics using ultra-wideband technology-based indoor navigation: A review. IEEE Access.

[22] Wang Z., Yang Z., Dong T. (2017). A review of wearable technologies for elderly care that can accurately track indoor position, recognize physical activities and monitor vital signs in real time. Sensors.

[23] Alarifi A., Al-Salman A., Alsaleh M., Alnafessah A., Al-Hadhrami S., Al-Ammar M.A., Al-Khalifa H.S. (2016). Ultra wideband indoor positioning technologies: Analysis and recent advances. Sensors.

[24] List of UWB-Enabled Mobile Devices. https://en.wikipedia.org/wiki/List_of_UWB-enabled_mobile_devices.

[25] Hu J.S., Wang J.J., Ho D.M. (2013). Design of sensing system and anticipative behavior for human following of mobile robots. IEEE Trans. Ind. Electron..

[26] Ferrer G., Sanfeliu A. (2019). Anticipative kinodynamic planning: Multi-objective robot navigation in urban and dynamic environments. Auton. Robot..

[27] Repiso E., Garrell A., Sanfeliu A. (2020). Adaptive side-by-side social robot navigation to approach and interact with people. Int. J. Soc. Robot..

[28] Karunarathne D., Morales Y., Kanda T., Ishiguro H. (2018). Model of side-by-side walking without the robot knowing the goal. Int. J. Soc. Robot..

[29] Jung E.J., Yi B.J., Yuta S. Control algorithms for a mobile robot tracking a human in front. Proceedings of the 2012 IEEE/RSJ International Conference on Intelligent Robots and Systems.

[30] Cifuentes C.A., Frizera A., Carelli R., Bastos T. (2014). Human–robot interaction based on wearable IMU sensor and laser range finder. Robot. Auton. Syst..

[31] Yan Q., Huang J., Yang Z., Hasegawa Y., Fukuda T. (2021). Human-following control of cane-type walking-aid robot within fixed relative posture. IEEE/ASME Trans. Mechatron..

[32] Nikdel P., Shrestha R., Vaughan R. The hands-free push-cart: Autonomous following in front by predicting user trajectory around obstacles. Proceedings of the 2018 IEEE International Conference on Robotics and Automation (ICRA).

[33] Conte D., Furukawa T. (2022). Autonomous Bayesian escorting of a human integrating intention and obstacle avoidance. J. Field Robot..

[34] Leica P., Roberti F., Monllor M., Toibero J.M., Carelli R. (2017). Control of bidirectional physical human–robot interaction based on the human intention. Intell. Serv. Robot..

[35] Young J.E., Kamiyama Y., Reichenbach J., Igarashi T., Sharlin E. How to walk a robot: A dog-leash human-robot interface. Proceedings of the 2011 RO-MAN.

[36] Scheidegger W.M., De Mello R.C., Sierra S.D., Jimenez M.F., Múnera M.C., Cifuentes C.A., Frizera-Neto A. A novel multimodal cognitive interaction for walker-assisted rehabilitation therapies. Proceedings of the 2019 IEEE 16th International Conference on Rehabilitation Robotics (ICORR).

[37] Honig S.S., Oron-Gilad T., Zaichyk H., Sarne-Fleischmann V., Olatunji S., Edan Y. (2018). Toward socially aware person-following robots. IEEE Trans. Cogn. Dev. Syst..

[38] Arechavaleta G., Laumond J.P., Hicheur H., Berthoz A. (2008). On the nonholonomic nature of human locomotion. Auton. Robot..

[39] Virtanen P., Gommers R., Oliphant T.E., Haberland M., Reddy T., Cournapeau D., Burovski E., Peterson P., Weckesser W., Bright J. (2020). SciPy 1.0: Fundamental Algorithms for Scientific Computing in Python. Nat. Methods.

[40] Brooke J. (1996). SUS—A quick and dirty usability scale. Usability Evaluation In Industry.

[41] Hart S.G. (2006). NASA-task load index (NASA-TLX); 20 years later. Proceedings of the Human Factors and Ergonomics Society Annual Meeting.

[42] Mathew J., Masson G., Danion F. (2020). Sex differences in visuomotor tracking. Sci. Rep..

[43] Kitson A., Riecke B.E., Hashemian A.M., Neustaedter C. NaviChair: Evaluating an embodied interface using a pointing task to navigate virtual reality. Proceedings of the 3rd ACM Symposium on Spatial User Interaction.

[44] Chang W.T. (2020). The effects of age, gender, and control device in a virtual reality driving simulation. Symmetry.

[45] Cherney I.D. (2008). Mom, let me play more computer games: They improve my mental rotation skills. Sex Roles.

[46] Nenna F., Gamberini L. The influence of gaming experience, gender and other individual factors on robot teleoperations in vr. Proceedings of the 2022 17th ACM/IEEE International Conference on Human-Robot Interaction (HRI).

